# Advantages and Recent Developments of Autologous Cell Therapy for Parkinson’s Disease Patients

**DOI:** 10.3389/fncel.2020.00058

**Published:** 2020-04-03

**Authors:** Teresia M. Osborn, Penelope J. Hallett, James M. Schumacher, Ole Isacson

**Affiliations:** Neuroregeneration Research Institute, McLean Hospital/Harvard Medical School, Belmont, MA, United States

**Keywords:** Parkinson’s disease, autologous, transplantation, dopamine neurons, cell therapy

## Abstract

Parkinson’s Disease (PD) is a progressive degenerative disease characterized by tremor, bradykinesia, rigidity and postural instability. There are approximately 7–10 million PD patients worldwide. Currently, there are no biomarkers available or pharmaceuticals that can halt the dopaminergic neuron degeneration. At the time of diagnosis about 60% of the midbrain dopamine (mDA) neurons have already degenerated, resulting in a depletion of roughly 70% of striatal dopamine (DA) levels and synapses. Symptomatic treatment (e.g., with L-dopa) can initially restore DA levels and motor function, but with time often lead to side-effects like dyskinesia. Deep-brain-stimulation can alleviate these side-effects and some of the motor symptoms but requires repeat procedures and adds limitations for the patients. Restoration of dopaminergic synapses using neuronal cell replacement therapy has shown benefit in clinical studies using cells from fetal ventral midbrain. This approach, if done correctly, increases DA levels and restores synapses, allowing biofeedback regulation between the grafted cells and the host brain. Drawbacks are that it is not scalable for a large patient population and the patients require immunosuppression. Stem cells differentiated *in vitro* to mDA neurons or progenitors have shown promise in animal studies and is a scalable approach that allows for cryopreservation of transplantable cells and rigorous quality control prior to transplantation. However, all allogeneic grafts require immunosuppression. HLA-donor-matching, reduces, but does not completely eliminate, the need for immunosuppression, and is currently investigated in a clinical trial for PD in Japan. Since immune compatibility is very important in all areas of transplantation, these approaches may ultimately be of less benefit to the patients than an autologous approach. By using the patient’s own somatic cells, reprogrammed to induced pluripotent stem cells (iPSCs) and differentiated to mDA neurons immunosuppression is not required, and may also present with several biological and functional advantages in the patients, as described in this article. The proof-of-principle of autologous iPSC mDA restoration of function has been shown in parkinsonian non-human primates (NHPs), and this can now be investigated in clinical trials in addition to the allogeneic and HLA-matched approaches. In this review, we focus on the autologous approach of cell therapy for PD.

## Overview of Dopamine Neuron Cell Therapy in Parkinson’s Disease and Advantages Over Current Available Therapies

### Current Therapies

**FIGURE 1:**
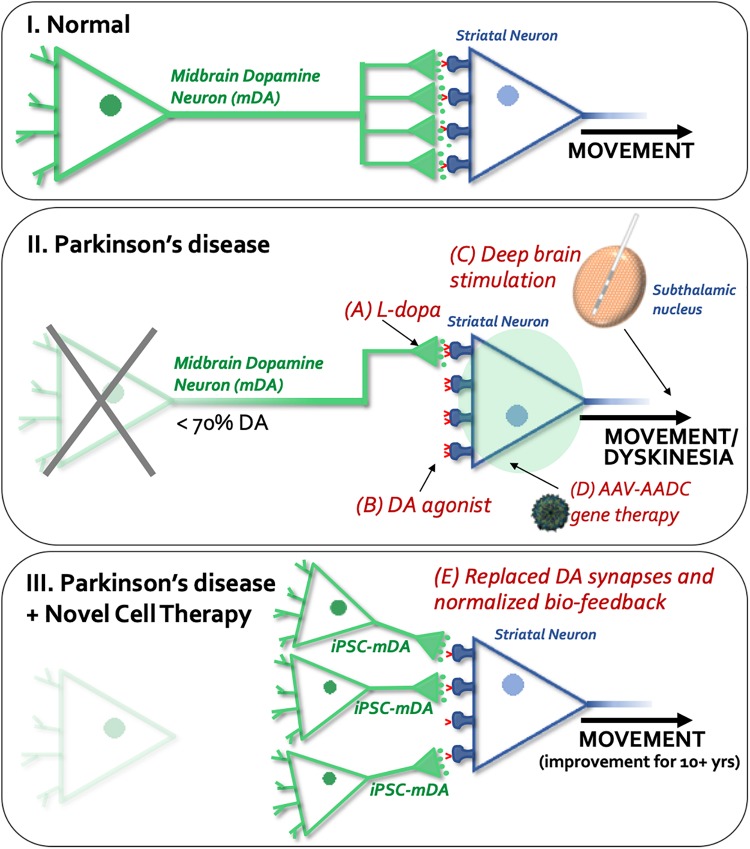
Schematic overview of available or experimental treatments and procedures for Parkinson’s disease. **(I)** Normal dopaminergic (shown in green) and striatal (shown in blue) synapses, and regulated dopamine (DA) release and reuptake (dopamine transporters shown in red) in the normal brain. **(II)** Shows current and experimental treatments: A, L-dopa; B, dopamine agonists; C) Deep brain stimulation (DBS), and D, gene therapy. The unique aspect of cell therapy (see **III**, E) is that it restores physiological dopamine release by synapses provided through new neurons implanted into the striatum.

The clinical movement disorder syndrome of Parkinson’s Disease (PD) occurs due to a progressive loss of midbrain dopamine (mDA) neurons. In fact, most patients remain free of clinical motor symptoms until the PD pathology has already reached an advanced stage with most (∼60%) of the selectively vulnerable dopamine (DA) neurons dysfunctional or dead, and with a consequent depletion of roughly 70% of striatal DA levels and synapses (Engelender and Isacson, 2017). For this reason, and since any retardation of degeneration is unlikely to be absolute, it is reasonable to develop cell replacement for the lost neurons. Such “live cell” replacement therapies are conceptually different from classical pharmacology. The current mainstay treatment for PD related motor symptoms is based on a pharmacological approach from 1957 using levodopa (L-dopa) (Carlsson et al., 1957) or dopaminergic agonists that elevate DA levels or stimulate DA receptors (Radad et al., 2005; Figure 1). Although this treatment can be effective for many years, its long-term and chronic use can result in the development of “motor complications,” including wearing-off, on-off fluctuations (Eriksson et al., 1984) and abnormal movements termed L-dopa-induced dyskinesias (Fahn, 2003). L-dopa crosses the blood-brain-barrier where it is converted to DA by dopa-decarboxylase containing cells; these include the remaining striatal dopaminergic terminals themselves, and also non-dopaminergic cells including cells in the blood-brain-barrier wall and serotoninergic neurons. Conversion of L-dopa to DA in non-dopaminergic cells following oral (non-continuous) administration of L-dopa, results in a pulsatile, non-physiological release of DA, which may act on supersensitive DA receptors in the striatum and contribute to the development of dyskinesias (Fahn, 2003). Newer dopaminergic agonists can activate DA receptors but they are not as effective as L-dopa, and in fact create substantial side effects on their own, including dyskinesia, albeit at a slower rate (Borovac, 2016). Deep brain stimulation (DBS) is another therapeutic modality but does not treat the DA neuron generation itself. DBS disrupts the circuits and functions as a depolarization blocker, which allows patients to take the same or higher L-dopa dose with less side effects, including dyskinesia, and dystonia (Herrington et al., 2016). It includes a surgical insertion of a medical device containing electrodes extending to the target region (subthalamic nucleus) and a pulse generator (Herrington et al., 2016) and carries the risk of major surgery. Generally, DBS works well initially but with time the circuitry readapts and over time is less effective in symptomatic relief (Buttner et al., 2019). In addition to the high initial surgical cost, patients require battery replacements every 3–5 years (Dang et al., 2019) and anticipating battery failure is also a critical clinical issue since it can result in a subacute worsening of symptoms (Montuno et al., 2013). Additionally, there is a risk that the DBS can result in cognitive side-effects if not implanted properly (Fields and Troster, 2000; Cernera et al., 2019).

### New Modalities

Other new modalities being explored for PD include gene therapy and cell therapy (Isacson and Kordower, 2008; Figure 1). Gene therapy creates enzymes to make L-dopa and DA non-synaptically (Mandel et al., 1999, 2008), similar to the use of a pump, without any cellular specificity or feedback control. From our and previous studies, gene therapy for neurotransmission defects are not likely to be helpful to the circuitry in patients as gene additions to striatal neurons will not control DA release.

Due to a lack of biofeedback and synaptic control in all current pharmacological therapies, they eventually lead to dyskinesias or other side-effects (see Figure 1). Therefore, there are efforts toward re-creating a synaptic DA release through the use of neural transplantation (Strecker et al., 1987; Clarke et al., 1988; Bjorklund et al., 2002; Isacson et al., 2003; Vinuela et al., 2008; Tsui and Isacson, 2011). To date, mDA cell preparations from aborted fetuses have been clinically tested and shown efficacy in PD patients (Lindvall et al., 1990; Freed et al., 1992; Kordower et al., 1998; Piccini et al., 1999; Hagell et al., 2000; Mendez et al., 2005; Redmond et al., 2008; Hallett et al., 2014; Kefalopoulou et al., 2014; Li et al., 2016). From a meta-analysis it was clear that if the fetal mDA cells are prepared and surgically injected appropriately, a 10–15 years of benefit is obtained with reductions in dyskinesia and off-time, and no additional side-effects appear (Barker et al., 2013). This improved function is because cell replacement using mDA neurons restores lost synapses. These new synapses functions with biofeedback regulation of DA locally in the synaptic microenvironment, resulting in physiological DA release and uptake, reducing the number of supersensitive DA receptors and providing long-term benefits for the patients with fewer side-effects (Figure 1, Vinuela et al., 2008; Tsui and Isacson, 2011). For these reasons and in a future perspective, cell therapy for PD when tested clinically to be safe and efficacious in moderate to severe patients, may potentially be used as a first-line of treatment to obviate the use of pharmacological DA therapies. When clinical trials using autologous or allogeneic midbrain neuron transplantation to PD patients are successful then cell therapy for PD would be a highly competitive treatment compared to currently available modalities.

**FIGURE 2:**
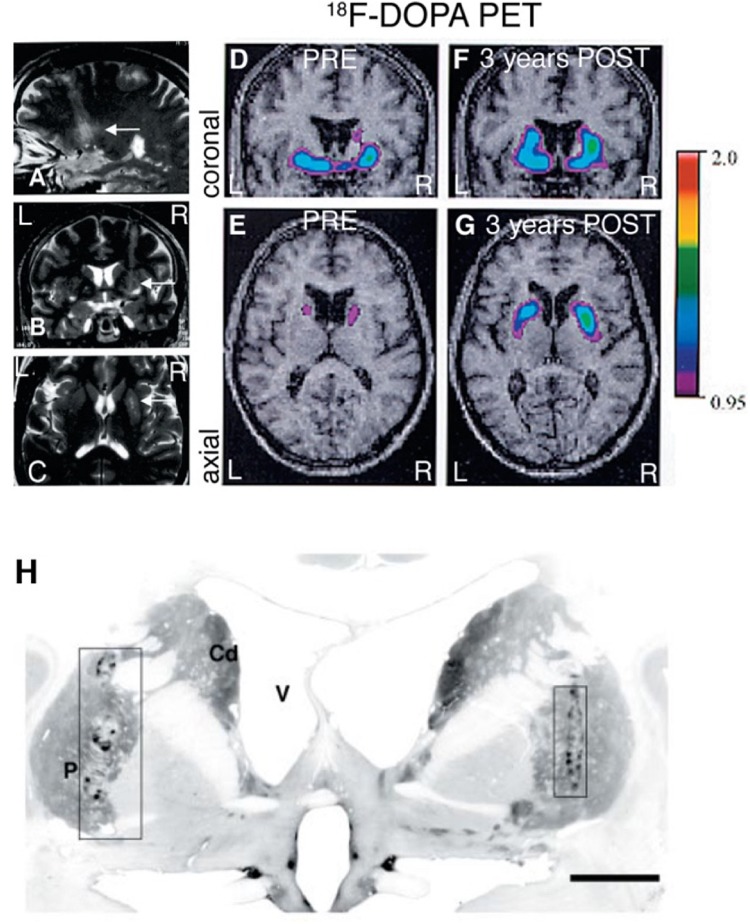
Fetal VM grafts. **(A–C)** MRI 24 h after fetal VM transplantation surgery in the right putamen showing the 4 needle tracks. **(D,E)** Preoperative PET scan showed a marked, asymmetrical decrease in putaminal18F-DOPA uptake, consistent with the diagnosis of idiopathic PD. **(F,G)** 3 years after transplantation the PETs show a significant increase in 18F-DOPA uptake. **(H)** TH immunostaining of the grafts 4 years after transplantation. (P = putamen; Cd = caudate nucleus; V = lateral ventricle. Scale bar: **H**, 1 cm). Figure originally published in Mendez et al. (2005). Reused by permission of Oxford University Press.

The cell-based therapy approaches in PD aim to replace nigrostriatal DA terminals lost by the disease process, with fetal or stem cell derived DA neurons placed directly into the caudate-putamen, and potentially also in substantia nigra. Cell replacement therapy with mDA neurons in PD addresses both the motor symptoms of PD, as well as L-dopa-induced dyskinesias. In the most successful cases (Mendez et al., 2005; Kefalopoulou et al., 2014), the requirement for L-dopa medication has been negated or substantially reduced. When new mDA neurons (which are autonomous pace-maker neurons, not needing afferent input to regulate transmitter release) are engrafted into the normal target regions of nigrostriatal DA neurons, they establish synapses with mature host striatal neurons and provide physiologically appropriate DA release and synaptic feedback control in the host brain (Zetterstrom et al., 1986; Vinuela et al., 2008). As discussed above, replacement of DA terminals in this manner may be far more effective in ameliorating the motor symptoms of PD than a pump-like pharmacological delivery of DA into the striatum that lacks physiological release and reuptake mechanisms (Figure 1). Long-term remarkable and neurologically clear benefits [approximately 50–60% reduction in Unified Parkinson’s Disease Rating Scale (UPDRS) scores off DA drug therapy] to PD patients following fetal DA neuron transplantation has been reported for over 18 years, including our own work (Mendez et al., 2005; Politis et al., 2010; Hallett et al., 2014; Kefalopoulou et al., 2014), and this outcompetes any current treatment for PD. Moreover, implanted fetal DA neurons can prevent progressive worsening of PD motor scores over at least 14 years (Kefalopoulou et al., 2014; Ivar Mendez, unpublished data). These clinical benefits are associated with evidence of physiological changes using PET and functional MRI neuroimaging (see Figure 2; Mendez et al., 2005). Our data also shows that transplanted fetal ventral mDA neurons remain healthy long-term (up to 14 years post-transplantation, the longest time-point we have studied to date) following transplantation into the putamen of PD patients, and despite ongoing disease processes in the host brain (Hallett et al., 2014).

## Emerging Options for Midbrain Dopamine Neuron Cell Sources

### Fetal Cells

Cell replacement therapy using DA neurons for PD in the clinic, has so far utilized ventral mDA neurons derived from fetal sources. More recently, attempts to replace missing DA neurons in PD preclinical models have evolved from this innovative and complex fetal DA cell transplantation method toward a potential scalable method that depends on stem cell-derived DA neurons (Hargus et al., 2010; Kriks et al., 2011; Sundberg et al., 2013; Grealish et al., 2014). The replacement using fetal neurons depended on the events of elective abortions providing fetal tissue of the midbrain including the dopaminergic neurons. This method was not scalable and, in most cases, only a few dozen patients were appropriately transplanted worldwide in monitored clinical trials (Barker et al., 2013). However, these conceptual advances of cell therapy for PD have helped accelerate the realization of regenerative medicine in PD.

### Induced Pluripotent Stem Cells and Embryonic Stem Cells

Induced pluripotent stem cells (iPSCs) provide several advantages over embryonic stem cells (ESCs) as a cell source for cell replacement in PD and other disorders, including the ability to use patient’s own cells, or HLA-matched cells and thus reduce (HLA-matched) or eliminate (autologous iPSCs) the need for any immunosuppression. Immune compatibility is universally known to be very important in all fields of transplantation. Immune suppression is not a trivial matter and may underlie some of the variability previously reported in clinical trials of fetal derived DA neuron transplantation (Barker et al., 2013). Indeed, it was previously reported that following the cessation of immunotherapy 6 months after fetal cell transplantation, PD patients lost the benefits of the transplantation (Olanow et al., 2003), and thus, a delayed immune or inflammatory response could have affected the long-term survival, growth, and function of the transplanted DA neurons. Currently there are plans to transplant dopaminergic neurons obtained from general human ES cells (allogeneic) into PD patients in clinical trials (Studer, 2017; Parmar et al., 2020). In addition, there has been an initiation of a clinical trial in Japan based on the work of Takahashi’s laboratory (Takahashi, 2017), where PD patients will receive HLA matched iPSC-derived dopamine neurons. The authors of this review will elect to test the fully autologous patient derived mDA neurons in clinical trials. The results of all of these studies will help guide the future approach for most benefit for the patients. Given how far along the PD field is in the steps toward developing sustainable cell therapy for PD, it is likely that what is learnt from these studies will also serve as a major milestone for cell and regenerative therapy for other parts of the brain and nervous system. Beyond PD, cell therapy as a modality can be used for other neuronal and glial disorders associated with the brain and the periphery (Freeman et al., 2000; Goldman et al., 2012; Cunningham et al., 2014).

### Universal Donor Cells

Another approach to avoid the need of immune suppression is the generation of a universal donor cell that will evade the immune system either by HLA engineering or immune cloaking strategies (Lanza et al., 2019). These approaches are developed from knowledge of how malignant and transmissible cancer cells evade the immune system or understandings of how pathogens and parasites have evolved to escape immune recognition (Lanza et al., 2019), and it is not fully known what potential safety issues the introduction of such changes in the cells might lead to. The specific risk with using HLA-negative cells is malignancies. Reduced HLA expression is a known mechanism with which cancer cells can evade the immune system and any transplanted cells turned cancerous could therefore be likely to go undetected by the immune system. Introduction of suicide genes that can be activated if cells turn malignant might be a safety strategy in the future (Lanza et al., 2019). However, autologous cells already provide a great system for naturally recognizing a dying or dysfunctional cell.

### Direct Conversion of Astrocytes

Another approach that would also eliminate the need of immune suppression is the direct conversion of somatic cells to DA neurons *in vivo* using virus technology. The current approaches for PD aim to convert astrocytes to DA neurons (Rivetti di Val Cervo et al., 2017). This could be a potentially interesting approach but is still in early exploratory stages. A potential pitfall of this strategy is the local loss of the astrocytes that are reprogrammed to neurons and the potential associated problems with this local astrocyte loss in a human brain. Astrocytes have numerous important functions, and many of these functions are essential for brain homeostasis and neuronal health. For example, they provide neurotrophic and metabolic support, regulate synaptogenesis and synaptic function, contribute to the blood-brain-barrier and play an important role in limiting the spread of local immune response initiated my microglia, preventing cell damage to surrounding tissue. There is also a cellular and molecular diversity among astrocytes, thus understanding what cells and functions are lost would be important to predict how a conversion of local astrocytes to DA neurons might affect the function of the brain in a PD patient (Khakh and Deneen, 2019).

## Early Efforts Toward Stem Cell-Based Cell Replacement Therapy for Parkinson’s Disease

**FIGURE 3:**
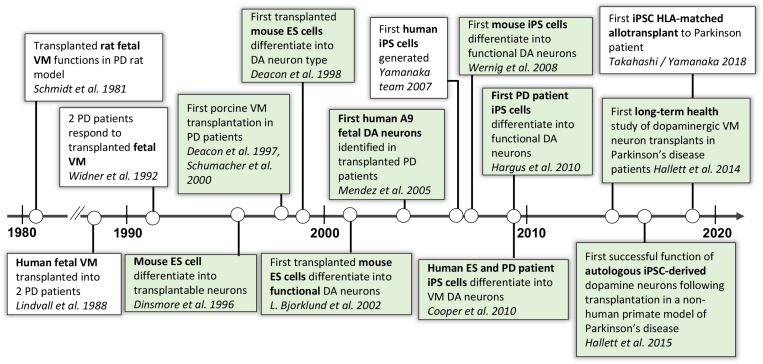
Progression of autologous cell therapy for Parkinson’s disease. In green are the discoveries and publications that have contributed to this timeline by the authors and their collaborators (Schmidt et al., 1981; Lindvall et al., 1988; Widner et al., 1992; Dinsmore et al., 1996; Deacon et al., 1997, 1998; Fink et al., 2000; Schumacher et al., 2000; Bjorklund et al., 2002; Mendez et al., 2005; Takahashi et al., 2007; Wernig et al., 2008; Cooper et al., 2010; Hargus et al., 2010; Hallett et al., 2014, 2015).

As described in Figure 3, our research team started an original stem cell-based cell therapy program for PD in 1998 (Deacon et al., 1998) and had by 2002 (Bjorklund et al., 2002) reached a point when mouse midbrain DA neurons could be derived from ES cells and work functionally in rodent models of PD. This work continued with the use of iPSCs, and in 2008 our team and collaborators published work on the first mDA neurons differentiated from mouse iPSCs and their function in PD animal models (Wernig et al., 2008), followed by mDA neurons differentiated from human iPSCs from healthy donors and sporadic PD patients in 2009, which also demonstrated functional effect in rodent PD animal models (Hargus et al., 2010).

## Proof-Of-Concept for Autologous Transplantation of Cynomolgus Monkey iPSC-Derived Midbrain Dopamine Neurons

**FIGURE 4:**
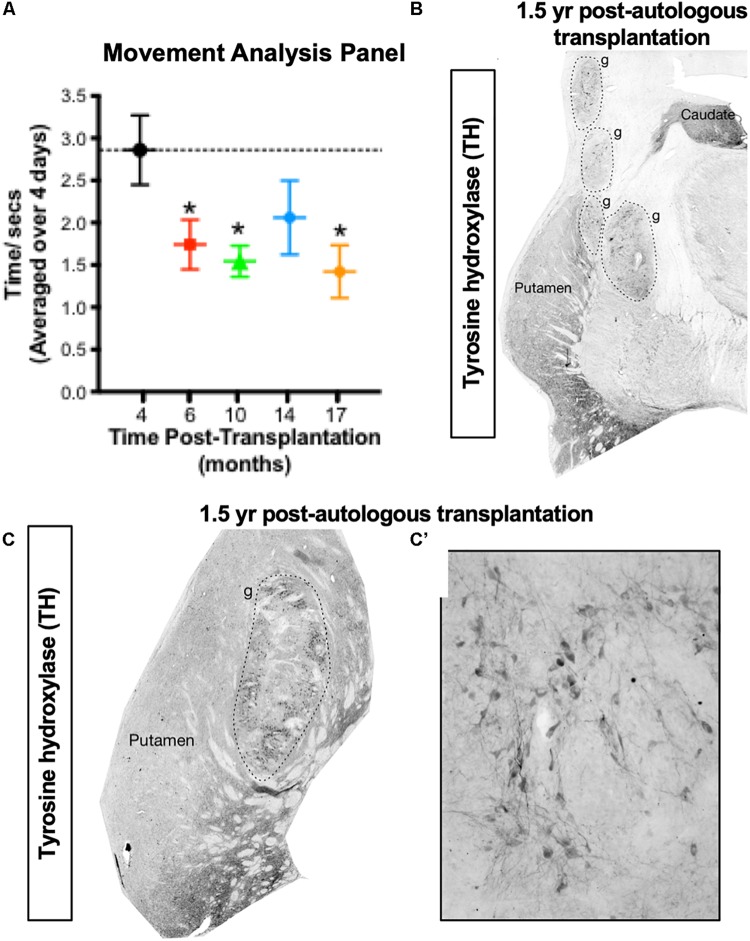
Proof of concept for the autologous transplantation approach in parkinsonian NHPs. **(A,B)** Autologous transplantation of iPSC-mDA cells into the left putamen of an MPTP-lesioned primate provides functional improvement in the right (contralateral) forelimb in an automated Movement Analysis Panel **(A)**, and survival of dopamine (TH+) neurons (>20,000) in the transplanted putamen **(B)**. **(C,C’)** Robust survival of TH + neurons at 1.5 years following autologous transplantation of iPSC-mDA cells into the left putamen of an additional MPTP-lesioned NHP (Osborn et al., 2020). These recent NHP data are supportive of our previous NHP findings (Hallett et al., 2015).

In 2015, our team published the first proof-of-concept (POC) data in non-human primates (NHPs) showing functional recovery and long-term survival of autologous transplanted iPSC-derived DA neurons. However, in this parkinsonian NHP model, unilateral autologous transplantation provided POC data for the long-term functional recovery of PD-like motor symptoms (increased daytime activity and reduction of time taken to complete a skilled motor task) for up to at least 2 years (Hallett et al., 2015). Behavioral improvement was accompanied by increased (compared to pre-transplantation values) DA transporter binding sites using PET neuroimaging, survival of engrafted DA neurons (>13,000), no inflammatory response, and no proliferating cells. Notably, no immunosuppression was administered to the recovered primate included in this study at any time, thus validating the use of autologous transplantation for use in clinical studies. In a recent study (Osborn et al., 2020) we have demonstrated functional restoration in two additional parkinsonian NHPs receiving autologous transplantation of iPSC-derived mDA cells (see Figure 4). In these animals, *even after 8 years of chronic PD without any spontaneous recovery, the parkinsonian NHPs improved functionally by the implanted iPSC-derived dopaminergic cells.* The degree of survival of the transplanted mDA neurons was consistent with our previously published work in primates (Hallett et al., 2015). From this work, we conclude that autologous transplantation provides functional recovery (reduced motor dysfunction) and transplanted tyrosine hydroxylase positive DA neurons survived in the putamen for at least 2 years after transplantation (the time of sacrifice). The additional confirmative data (see Figure 4) showed that functional recovery was observed with ∼20,000 surviving tyrosine hydroxylase positive neurons in the graft. There was no observable immune response present, as assessed by staining for microglia in the graft and in the neighboring host putamen. Consistent with these data, a recent study using xeno-grafting of human iPSCs into parkinsonian NHPs, demonstrated that a similar number of surviving DA neurons (∼16,000) resulted in functional improvement of the immunosuppressed primates, validating our present and previous reports of the effectiveness of this cell-dose in NHPs (Kikuchi et al., 2017).

## Methods Relevant for Generating Midbrain Dopamine Neurons From Induced Pluripotent Cells for Autologous Transplantation

### mDA Neuron Differentiation

**FIGURE 5:**
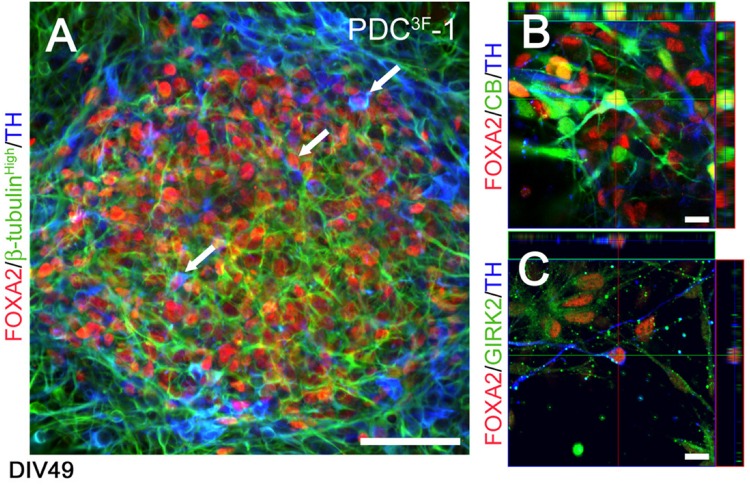
Phenotypic characterization of FOXA2 + dopaminergic neurons generated by the differentiation protocol described in Cooper et al. (2010) at DIV49. **(A)** Human iPSCs generate FOXA2 + (red) dopaminergic neurons (TH, blue; β-tubulin, green; arrowheads). **(B,C)** Cells co-expressed TH (blue), FOXA2 (red) and calbindin (green, B) or GIRK2 (green, C), indicative of an A10 or A9 DA neuron phenotype, respectively. Scale bar **(A)** = 50 μm, **(B,C)** = 10 μm. Reprinted from Cooper et al. (2010) with permission from Elsevier.

iPSCs, when exposed to a mixture of small molecules, can be differentiated toward mDA neuron fate (Figure 5; Cooper et al., 2010). Protocols published prior to 2010 (Roy et al., 2006; Sonntag et al., 2007; Chiba et al., 2008; Yang et al., 2008) resulted in a very low fraction of the A9 mDA neuron subpopulation that originates in the substantia nigra pars compacta and is lost in PD patients. A comprehensive study was published in 2010 (Cooper et al., 2010) that discerned factors important for the generation of this DA neuron subpopulation. Careful titrations of retinoic acid levels show that 10nM retinoic acid significantly improved the expression of the transcription factor (TF) Engrailed-1, a TF important for mDA development and survival (Cooper et al., 2010). Additional changes to previously published protocols (Sonntag et al., 2007; Cai et al., 2009) included a more potent form of sonic hedgehog recombinant protein, specification of the subtype of FGF-8 used (FGF-8a rather than FGF-8b) and the use of recombinant Wnt-1 for canonical Wnt pathway (Wnt/β-catenin pathway) activation. These changes generated a human neural progenitor cell population that exhibited a transcriptional profile (Muhr et al., 1999; Nordstrom et al., 2002, 2006) consistent with midbrain regionalization (Cooper et al., 2010). Several protocols are now available worldwide for midbrain differentiation that includes DA neurons (Cooper et al., 2010; Kriks et al., 2011; Sundberg et al., 2013; Kikuchi et al., 2017; Nolbrant et al., 2017) and protocols are being refined by use of xeno-free procedures and highest grade available reagents (Nolbrant et al., 2017; Osborn et al., 2017) in order to improve reproducibility of differentiations and eliminate components incompatible with human transplantations (Cooper et al., 2012). These protocols differ in a few specific aspects (Cooper et al., 2010; Kriks et al., 2011; Sundberg et al., 2013; Kikuchi et al., 2017; Nolbrant et al., 2017) and as described below some teams chose to use progenitor cells whereas others prefer the post mitotic equivalence of fetal neurons.

### Maturity of Transplantable Cells

**FIGURE 6:**
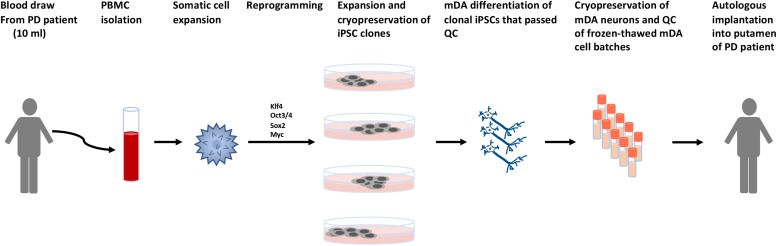
Schematic describing the process for autologous cell therapy for Parkinson’s disease. Blood is collected from patients by venipuncture. Peripheral blood mononuclear cells (PBMCs) are isolated and the specific somatic cell population for reprogramming is expanded. Reprogramming factors (Takahashi et al., 2007) are introduced and clonal lines expanded for quality control testing. Clones that pass all quality control (QC) steps are used for mDA differentiations. The mDA neurons are cryopreserved and the batches quality control tested prior to autologous transplantation.

The decision of whether to transplant the mDA patterned cells at a progenitor stage, as post-mitotic neurons or a mix thereof determines what cell type markers are used in the quality control process. Furthermore, in order to allow for quality control testing of cell batches for transplantation and allow flexibility as to when and where a patient undergoes the transplantation surgery the cells need to be cryopreserved. The maturity and timing selected for cryopreservation is also important as it can impact the survival of the dopaminergic neurons. If the cells are frozen at too mature of a stage, the survival of the DA neurons after transplantation is less than if frozen and transplanted at an early post-mitotic stage or progenitor stage (Osborn et al., 2015). Different groups are using different approaches and timing for freezing (Kriks et al., 2011; Nolbrant et al., 2017; Osborn et al., 2017), which in turn leads to different markers being relevant for use in pre-transplantation criteria (Kee et al., 2017; Kirkeby et al., 2017; Osborn and Hallett, 2017). Tyrosine hydroxylase (TH) staining is an indicator of post-mitotic dopaminergic neurons that in recent protocols start to be expressed at about day *in vitro* 17. Therefore, it can be used together with FoxA2 as an indicator of midbrain dopaminergic neurons and the percentual co-positivity of the two can be set as a positive cellular marker criterion for mDA cell transplantations. In cases where only progenitor cells are transplanted one must rely on additional markers to determine dopaminergic progenitors and predict future dopaminergic cell content (Kee et al., 2017; Kirkeby et al., 2017; Osborn and Hallett, 2017). Given that several different preclinical studies have been successful in transplanting at various time-points (DIV16-49) (Hargus et al., 2010; Kriks et al., 2011; Sundberg et al., 2013; Doi et al., 2014; Hallett et al., 2015; Kikuchi et al., 2017; Nolbrant et al., 2017; Osborn et al., 2017; Wakeman et al., 2017) and resulting in similar functional grafts containing mDA neurons, it is premature to say what is the most optimal protocol and strategy. Of note, the only grafted cells that have shown to generate functional recovery in parkinsonian NHPs have been with cell preparations that contain post-mitotic neurons (Hallett et al., 2015; Kikuchi et al., 2017). Furthermore, although mDA neurons are the cell type that is responsible for the functional effect in grafts, the midbrain cell population produced does not necessarily require cell sorting since all clinical experience to date, using fetal cell transplantation, includes a mixture of midbrain cells. It is in fact possible that removing the other midbrain companion cells may reduce trophic interactions necessary (positive bystander effect) for substantia nigra survival (Hedlund et al., 2008). The autologous approach as planned by this team is summarized in Figure 6. The future pre-clinical and clinical studies, on-going and planned will provide a guide of the specifics for the most efficacious and safe cells or cell compositions for transplantation, whether pure mDA neurons or a mixed midbrain cell composition and whether progenitors, post-mitotic neurons or a mixture of both are preferred.

## Prospective Long-Term Health of Transplanted Dopamine Neurons

A misconception associated with using cell replacement therapy in neurodegenerative disorders, is that the transplanted cells will eventually succumb to the same pathological processes and disease that presented in the host brain, resulting in reduced function of the transplanted cells. In the PD field, this is based on popular theory about pathological “spread” of α-synuclein from host to transplant (Kordower et al., 2008; Li et al., 2008), and the observations that a low percentage of transplanted fetal dopaminergic neurons contain α-synuclein immune reactive inclusions over a decade after transplantation (Kordower et al., 2008; Li et al., 2008, 2016). Discrepancies in success of fetal dopaminergic neurons come from procedural differences in these transplantations (Redmond et al., 2008; Cooper et al., 2009). When the fetal tissue is dissociated to a cell suspension prior to transplantation, the grafts remain healthy 14 years post-transplantation and have none or very few α-synuclein inclusions after 14 years (Mendez et al., 2008; Hallett et al., 2014). However, if transplanting cellular aggregates instead of cells in suspension, the grafts are surrounded by activated microglia (Li et al., 2008; Kurowska et al., 2011) and around 2–12% of the dopaminergic neurons in the grafts have α-synuclein positive inclusions after 12–24 years (Kordower et al., 2008; Li et al., 2008, 2016). Importantly, there is no evidence of any clinical or functional relevance of such limited pathology (Cooper et al., 2009). In fact, transplanted fetal midbrain dopaminergic neurons have been shown to function, as evidenced by improvements in PD motor symptoms, for over a decade after transplantation (Li et al., 2016) with the longest documented functional improvement for at least 18 + years (Hallett et al., 2014; Kefalopoulou et al., 2014). In fact, even at these long time frames, patients have been able to reduce or discontinue pharmacological DA replacement therapy (Kefalopoulou et al., 2014). Another way of looking at this issue in a medical and biological perspective is to raise the simple functional question that if the transplants really were under “attack,” this would be contradicted by the clinical facts of striking clinical benefits that cell replacement of mDA neurons can provide. Using cell therapy, it is surprising but true that transplanted neurons can remain functional for at least 10–20 years and show no histological evidence or neuritic pathology. These cell therapy clinical studies have had no gene modifiers, including of α-synuclein, or any or blocking of α-synuclein function (Hallett et al., 2014; Kefalopoulou et al., 2014). Therefore, the planned clinical trials by several groups worldwide are aligned on the scientific and biological view that newly implanted functional DA neurons are not affected significantly or functionally by the underlying disease process at least for several decades (Astradsson et al., 2008). Moreover, our data and others studying human cells transplanted to patients with PD, provides a perspective of the actual development of clinical PD in the patient’s own DA cells (Hallett et al., 2014) which do not succumb to dysfunction and detrimental pathology until in the vast majority of cases, at least in the 6th–7th decade of life.

## The Use of Autologous Cells With Possible Genetic Predisposition to Disease

The majority of patients with PD (>85%) have sporadic forms, and the genetic forms of PD are relatively rare. However, even in cases where the patients have underlying cellular problems due to genetic variants, they have surviving functional DA neurons for the majority of their life, in some cases up to >60 years of age, before any noticeable degeneration that results in functional impairment occur. Even in severe familial genetic cases (for example, α-synuclein mutation, or copy number variants), PD does not present for 30 years or more. Therefore, it is both logical and reasonable to assume that even the most severe, rare genetic forms would, after transplantation of new young cells, have highly adaptive functional DA neurons for synaptic signaling for at least 20–25 years. In fact, both rodent (Hargus et al., 2010) and NHP (Kikuchi et al., 2017) studies have demonstrated that human iPSC-derived mDA cell preparations from both healthy subjects and PD patients work equally well in restoring synapses and motor function in parkinsonian animal models. On the contrary, long-term restorative function has not yet been shown in parkinsonian NHPs for mDA neuron preparations differentiated from human ES cell lines, despite significant efforts.

## Advantages of Autologous Cells for Transplantation in Pd Over Allogeneic or MHC-Matching

There are several advantages of the autologous cell therapy approach over allogeneic or MHC matching (Emborg et al., 2013; Morizane et al., 2013; Hallett et al., 2015; Table 1). One obvious advantage, relative to allogeneic cell transplants, is that autologous iPSC can be used in PD patients without the need for immunosuppression (Hallett et al., 2015). The question about the need for immunosuppression is important at many levels:

**TABLE 1:** Adavantages of autologous transplantation for Parkinson’s disease.

• Autologous approach requires no immune suppression in patients (as also demonstrated by the team’s POC non-human primate data).
• Competing allogeneic and MHC-matched cell therapy approaches will require long-term (6–12 + months) immune suppression. Systemic immune suppression, as required for MHC-matched and allogeneic approaches is not trivial in older or frail patients, and some patients may not be able to tolerate it and will have significant morbidity.
• There are significant immunological effects of allogeneic transplants.
• Autologous neural cell transplants potentially are better integrated and have axonal networks and better functional effects than non-autologous transplants. Even modest immune activation is expected to be detrimental to graft function and synaptic connectivity.
• The medical transplantation field, hospitals, health care providers, and payors of health care have learned significantly from decades of autologous vs. allogeneic cell therapy in patients requiring bone marrow transplantation. Some of this learning may apply to autologous cell therapy for brain degenerative diseases like Parkinson’s disease.

(1) *Basic biology of cell integration and recognition:* Autologous neural cell transplants potentially are better integrated, have better axonal networks (Emborg et al., 2013; Hallett et al., 2015) and have better functional effects than non-autologous transplants (Hallett et al., 2015). A relatively low number of autologous dopaminergic midbrain neurons derived from iPSCs (∼13–14,000) can be sufficient to reverse parkinsonism in NHPs (Figure 4; Hallett et al., 2015; Osborn et al., 2020).
(2) *Rejection by immunological mechanisms at cellular and synaptic levels*: A primate study demonstrated clear benefits to the use of autologous transplants rather than allografts in both cell survival and immune response (Morizane et al., 2013). A different primate study looking at both the acute and subacute immune response showed that MHC matching improves the engraftment of iPSC-derived mDA neurons in NHPs. But although MHC matching reduced the immune response, it did not completely prevent an immune reaction and the conclusion was that MHC matching still needs to be combined with immunosuppressive drugs but MHC matching might reduce the required dose and duration of such therapy (Morizane et al., 2017). Studies with fetal mDA neurons in the clinic have demonstrated several cases in which the clinical beneficial response is reduced after removal of immune suppression by 6–9 months after cell implantation (Olanow et al., 2003). Decades of studies of allogeneic brain transplantation demonstrates sensitization of the B cell component of the immune system (Kordower et al., 1997), providing increasing antibody titers to such allogeneic transplants. Clearly, the brain immune system is capable of activating both T and B cell responses to interfere with neuronal transplants (Kordower et al., 1997). Should future trials of allogeneic transplants show variable rejections, it will be an enormous obstacle to remove immune suppression or scale to a larger group of patients, as patients would not want to risk transplant rejection and loss of function. Attempts to simplify an allogeneic universal cell source by removing HLA antigens and related MHC systems may occur in the future, but there is almost nothing known about the need for the immune system to eliminate unhealthy or dying cells that are genetically manipulated in such a way. The advantage of autologous cell sources is that the natural biology for cell/transplant integration, recognition and function is coupled with an immune competence to eliminate dying or dysfunctional cells, as would occur normally in any brain or biological tissue. Given this, autologous cell therapy may become a gold standard for which the other future cell therapies need to be measured; and
(3) *The health risks to the patient recipients*: Patients with compromised immune system are always considered high risk recipients for systemic immune suppression for any amount of time. Immune responses as observed with allografting or xenografting can be detrimental to transplant function (Soderstrom et al., 2008; Morizane et al., 2013). Data from already well-established medical disciplines demonstrate significant differences in risk profiles between autologous and allogeneic transplants. An illustration is the relevant stem cell bone marrow transplantation therapy, where the morbidity, mortality, length of hospital stays and costs are reduced by autologous approaches (Rowe et al., 1994; Majhail et al., 2013). Taken together, autologous transplantations overcome several limitations as described above, which will likely lead to improved outcomes in many scenarios, including risk for graft-host rejections, local immune responses that clearly reduces functional synaptic transmission, and morbidity risk for patients taking severe immunosuppressive drugs.

## Consideration of Healthcare Costs and Benefits Relative to New Cell Therapies

A conventional view, criticism and perception is that autologous cell therapy is always more expensive than allogeneic cell therapy. It is true that an allogeneic or HLA-matched approach would allow for larger batches of cells to be produced and quality control tested for use in multiple patients, whereas an autologous cell transplantation approach requires preparation of cell batches for each patient and quality control testing of each batch, which initially (but less so with increased scale and automation) drives up the cost of the cell production step. However, when looking at the total healthcare cost, this conventional view is not necessarily true (Majhail et al., 2013). An interesting future perspective is that many payors may try to avoid allogeneic transplants given the documented higher current costs due to immune suppression and transplant rejection. In addition, as outlined above, for neural transplantation and maybe other cell types, the functional integration is improved with recognition of autologous antigens. In well-established medical disciplines there are significant differences in risk profiles between autologous and allogeneic transplants. An example is stem cell bone marrow transplantation cell therapy, where the morbidity and costs for failed allogeneic transplants is much higher than for autologous transplants (Majhail et al., 2013). The medical transplantation field, hospitals, health care providers, and payors of health care have learned significantly from decades of autologous vs. allogeneic cell therapy in patients requiring bone marrow transplantation. Some of this learning may apply to autologous cell therapy for brain degenerative diseases like PD. For bone marrow stem cell transplantation, payors and health care providers can estimate over $100,000 per patient for completed *autologous* bone marrow transplantation cell therapy and follow-up. However, the average estimation for payor and health care costs for *allogeneic* transplants and cell therapy can be several-fold higher (Majhail et al., 2013). The reason is that allogeneic transplantation presents a large and significant morbidity risk to the patients due to immunological and prominently immune suppression issues (Rowe et al., 1994). The high risk for patient morbidity creates a significant burden and additional cost to the health care system, where the potential very large cost per patient of allogeneic cell therapy needs to be viewed as a potential loss of benefit to those who could receive an effective autologous transplant. For payors and the healthcare system the average expected cost of allogeneic transplants is therefore in reality higher than autologous cell therapy. Whether these considerations also apply to allogeneic vs. autologous transplantation of brain cells into the CNS will hopefully become evident early in the process of safety/Phase 1 trials in humans. With this future perspective in mind, we believe there are several reasons why overall, the health care systems will likely support technical and medical innovation that support autologous transplants for most applicable medical conditions. In such a perspective, this overall strategic support for autologous transplantation will also apply to autologous cell therapy approaches for PD and related disorders.

## Author Contributions

TO, PH, JS, and OI wrote this manuscript.

## Conflict of Interest

The authors declare that the research was conducted in the absence of any commercial or financial relationships that could be construed as a potential conflict of interest.
